# Equivalent circuit parameters of planar transmission lines with spoof surface plasmon polaritons and its application in high density circuits

**DOI:** 10.1038/s41598-019-55200-z

**Published:** 2019-12-11

**Authors:** Chia Ho Wu, Linfang Shen, Hang Zhang, Jinhua Yan, Da Jun Hou, Guobing Zhou, Yu Lin Wu

**Affiliations:** 1 Department of Applied Physics, Zhejiang University of Technology, Hangzhou, 310023 China; 2 Institute of Electro-Optical Science and Technology, National Taiwan Normal University, Taipei, 11677 Taiwan

**Keywords:** Nanophotonics and plasmonics, Nanophotonics and plasmonics, Nanophotonics and plasmonics

## Abstract

In this paper, the characteristics of a class of transmission lines which support spoof surface plasmon polaritons are investigated both numerically and experimentally. In order to provide the characteristic impedance of spoof surface plasmon polaritons for PCB designers, the equivalent circuit parameters of the microstrip line periodically structured on subwavelength scale are extracted with the numerical method. It is found that the equivalent circuit parameters significantly vary with frequency when the subwavelength periodic structure is introduced into the edge of the conventional microstrip line. The results of time-domain measurements show that spoof surface plasmon polaritons have remarkable advantage over conventional microstrip lines and can be directly used in actual high-speed circuits, which is helpful for eliminating the doubts whether the metamaterials can be directly used in actual circuits.

## Introduction

Novel physical phenomenon induced by interaction between electromagnetic (EM) field and subwavelength metal structure has attracted much attention, and lots of peculiar devices have been demonstrated which greatly improve the circuit and antenna performances. In 2004, J. B. Pendry proposed a new mechanism of surface wave in metal for microwave (or terahertz) frequencies^1^, where the metal surface is periodically structured on subwavelength scale by drilling holes in it. Such a surface wave is referred to as spoof surface plasmon polariton (SSPP), as its dispersion property is similar to conventional SPPs at visible frequencies. But the properties of SSPP are only determined by the geometric parameters of surface structure. It is known that SPPs are EM excitations on the interface between metal and dielectric. The fields of SPPs peak at the interface and decays exponentially away from it in the metal and dielectric, and they can be highly confined on subwavelength scale^2^. As a result, optical waveguide devices can be microminiaturized based on SPPs^3^, and this is essentially important for photonic integrated circuits and subwavelength optical devices^4^. Several types of plasmonic waveguides for digital photonic chips were discussed in detail^5^. It was also reported that SPPs can effectively stimulate Raman optical active and be used to reveal the absolute molecular conformation^6^. SPPs are finding an ever increasing number of applications in traditional domains and newly emerging nano-photonic and optoelectronic technologies.

However, as the plasma frequencies of metals mostly locate in the ultra-violet (UV) regime, metals behave as perfect electric conductor (PEC) at low frequencies (such as in terahertz or microwave regime), and they cannot sustain SPPs with high confinement there. SSPPs are related to an effective plasma frequency of a structured metal surface, which can be tuned with geometric parameters^7,8^. The physical mechanism of SSPPs was discussed in^9^. There are distinct differences between SSPPs and conventional SPPs. The former exist in the subwavelength metal periodic structure, so its propagation characteristic is strongly dependent on the geometric parameters. The transmission frequency range of SSPPs is determined by the lattice constant as well as the shape of the unit cell in the periodic structure. The advantage of SSPPs is evident in many waveguide structures. In^10^, a kind of single metallic corrugated transmission line was reported to support highly confined terahertz (THz) waves. THz and microwave devices based on SSPP were proposed and studied in^11–16^. Afterwards, leaky-wave antennas based on SSPPs were demonstrated in the microwave regime^17,18^. In addition, Gao *et al*. proposed a way to controll SSPPs by using photonic crystal effect, which provides high flexibility for SSPP waveguide design^19^.

It is known that the mutual coupling between adjacent parallel microstrip lines becomes serious when working frequency increases, and this would drastically influence the performance of the entire circuit^20^. Several schemes have been proposed to suppress the coupling effect. Serpentine guard trace was introduced between parallel microstrip lines^21^, however, extra capacity effect caused by a large number of grounded wires increases the rising time of signal and decreases the transmission rate. The differential microstrip line can reduce mutual coupling and is widely used in integrated circuit^22^. Nevertheless, the mutual coupling is still severe in densely distributed differential microstrip lines, and the differential signal will be converted to common mode signal under the influence of the surrounding environment. Obviously, new physical concept is necessary to overcome such mutual coupling, and so far SSPPs seem to be a good candidate.

Recently, the concept of SSPPs has been applied in a planar integrated circuit to suppress the coupling effect between microstrip lines^23^. In the actual situation, however, if the subwavelength periodic microstrip line is utilized to improve the performance of printed circuit board (PCB) circuits, its equivalent circuit parameters (such as capacitance, inductance, resistance, conductance per unit length, and characteristic impedance) is not known yet, which is necessary for PCB designers. Only based on these parameters with enough accuracy, one can establish an equivalent circuit model of interconnect lines, then the related system combined with active devices can be analyzed with the circuit theory. For this purpose, in this paper, the capacitance, inductance, resistance and conductance of microstrip lines with subwavelength periodic corrugations (SPCs) are numerically calculated with the finite element method according to their basic definitions. Then the impedance of the subwavelength periodic microstrip line is further evaluated, and it is compared with the corresponding results from the time domain reflectometer (TDR) measurement. To illustrate the possible application of such new type of microstrip line in high-speed PCBs, the transmission characteristic of the structured microstrip lines is experimentally analyzed with time domain signals. It is found that the subwavelength periodic microstrip line exhibits much better performance than the conventional microstrip line.

## Results

### Equivalent circuit analysis

**Figure 1:**
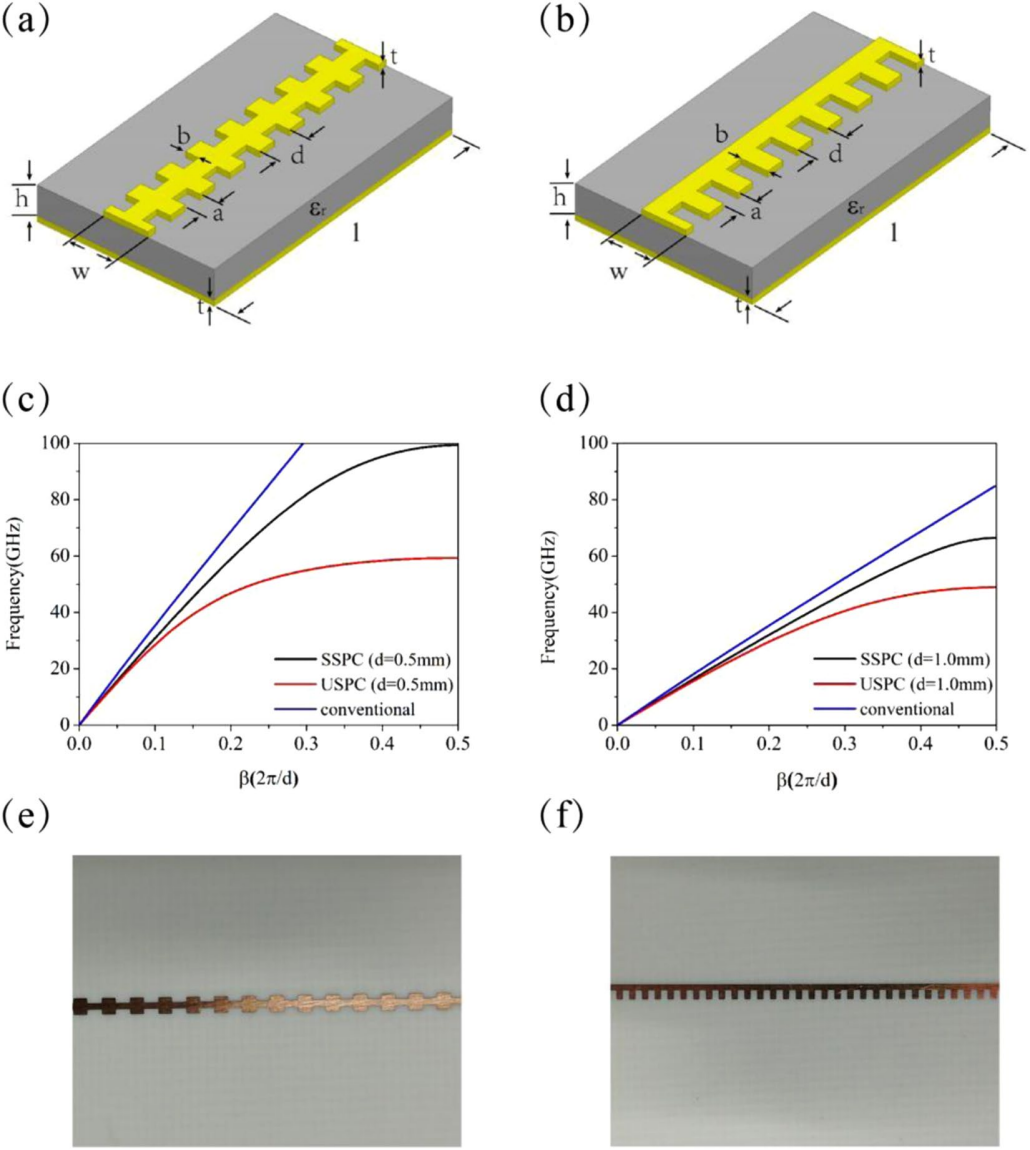
(**a,b**) Schematics of microstrip lines with BSPCs or USPCs. (**c,d**) Dispersion relations for the microstrip lines with BSPCs and USPCs for two different periods. The dispersion relation for the conventional microstrip line is also included in (**c,d**) for comparison. (**e,f**) Optical images of the samples for (**a,b**).

Two types of microstrip lines periodically corrugated with subwavelength periods are studied in this paper. Figure 1(a,b) show the schematics of the two microstrip lines, and Fig. 1(e,f) are the optical images of their samples. The microstrip line of the first type has grooves on both sides of the strip [called bilateral subwavelength periodic corrugations (BSPCs)], which is characterized by the geometric parameters as follows: the line width *w*, lattice constant *d*, groove depth *b*, groove width *a*, substrate dielectric constant *ε*_*r*_, substrate thickness *h*, and metal thickness *t*. In order to make SSPPs match with the actual circuit in impedance, a metal film (such as Cu) is added to the bottom of the dielectric layer as the ground plane. The width of the groove is a half of the lattice constant, i.e. *a* = 0.5*d*. The the microstrip line of the second type, which has unilateral subwavelength periodic corrugations (USPCs), has the same line width *w* as the one with BSPCs.

To investigate the transmission properties of microstrip line with SPCs, we calculate the propagation constant (β) of SSPPs with finite element method. Due to the periodicity of the structure, the propagation constant is limited to the first Brillouin zone, i.e., |*β*| ≤ *π*/*d*. The lattice constant *d* is chosen to be $d\ll \lambda$, thus no bandgap will appear in the frequency range of our interest. For the experimental samples, we choose RO4003 substrate with *h* = 0.508 mm, *ε*_*r*_ = 3.37, and *t* = 0.0175 mm. The width of the stripe lines of both considered types is taken to be *w* = 1.2 mm. For the conventional stripe line with *w* = 1.2 mm, the characteristic impedance has a theoretical value of 48.54Ω, and our TDR measured result is 47.69Ω, which has a relative error of 1.75%. It was reported that corrugated microstrip lines can achieve coupling suppression even for a small width of *w* = 0.24 mm^24^.

**Figure 2:**
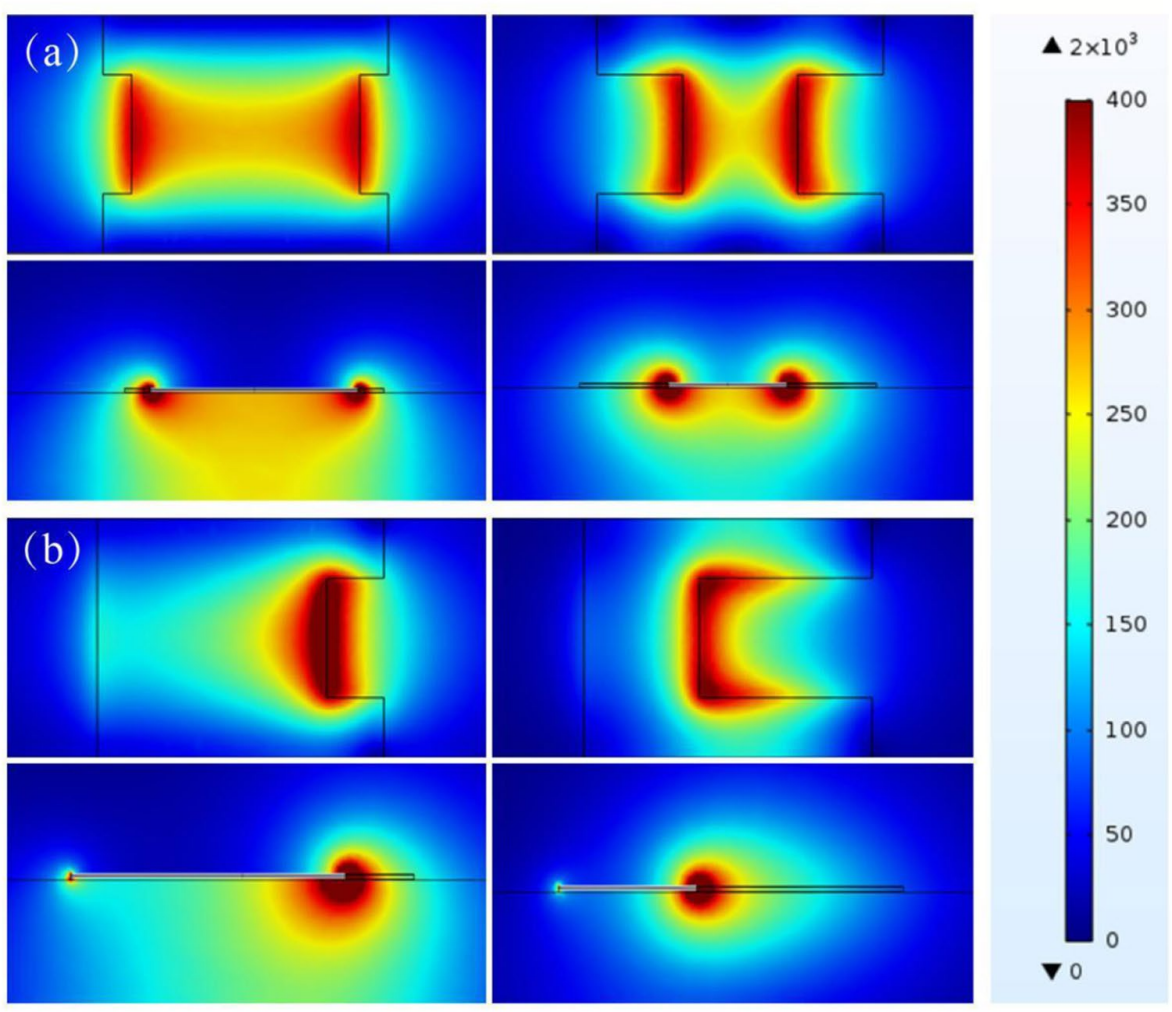
Magnetic field distributions on different cutting slices for the microstrip lines with BSPCs (**a**) and USPCs (**b**). In both (**a**) and (**b**), the upper two panels are the horizontal slices just below the corrugated metal line, and the lower two panels are the vertical slices halving the groove. In (**a**), *b* = 0.1*w* for the left panels and *b* = 0.3*w* and right panels. In (**b**), *b* = 0.2*w* for the left panels and *b* = 0.6*w* for the right panels.

We calculated the dispersion relation for the proposed subwavelength periodic microstrip lines. Figure 1(c) shows the dispersion curves for the microstrip lines with BSPCs and USPCs, with the lattice constant *d* = 0.5 mm, and groove depth *b* = 0.3*w* and *b* = 0.6*w*. It is found that the asymptotic frequencies are *f*_*s*_ = 99.3 GHz and 59.34 GHz for the BSPC and USPC, respectively. For comparison, the dispersion curve for the conventional microstrip line is also plotted. Apparently, working frequency bands are different for the two microstrip lines with different unit cell shapes. We will reveal that the two microstrip lines have completely different levels of coupling suppression effect. Figure 1(d) shows the dispersion curves for the two microstrip lines in the case of *d* = 1.0 mm, where the asymptotic frequencies are *f*_*s*_ = 66.453 GHz and 48.92 GHz for the BSPC and USPC, respectively. The asymptotic frequencies for BSPC and USPC can be further reduced to *f*_*s*_ = 36.44 GHz and 32.96 GHz when *d* = 2.0 mm. The working bandwidth of the proposed microstrip line is determined by the asymptotic frequency. Evidently, when the lattice constant *d* increases, the transmission bandwidth decreases, and the bandgap above the asymptotic frequency may occur in the working frequency range for the high-speed circuit. As a result, the bandgap directly influences the property of transmitted signal. Figure 2 shows the magnetic field distributions on the horizontal and vertical slices at the asymptotic frequencies for the two microstrip lines with different groove depths. For each type of microstrip line, the the field of SSPPs is confined more effectively for the case of deeper groove. On the other hand, the shape of the unit cell in the structured microstrip line obviously influences the its transmission property, the one with BSPCs has a larger bandwidth than the other with USPCs.

To apply SSPPs in actual circuits, the equivalent circuit parameters of the subwavelength periodic microstrip lines must be clarified, such as the inductance *L*, capacitance *C*, resistance *R*, and conductance *G* per unit length, and the characteristic impedance *Z*_0_, which can be used to describe the transmission property of the microstrip line. For periodically textured transmission lines, there were some attempts to evaluate equivalent circuit parameters in^25,26^. It is seen that in the low-frequency limit of $d\ll \lambda$ (where *d* is the lattice constant and *λ* the vacuum wavelength), the phase velocity and characteristic impedance of the periodic transmission line is very close to the values of a uniform transmission line, which are given by

1 $${v}_{p}\cong 1/\sqrt{LC},$$

2 $${Z}_{0}\cong \sqrt{\frac{L}{C}}$$

where *C* and *L* are the capacitance and inductance per unit length. The capacitance *C* and inductance *L* per unit length of our structured microstrip lines will be extracted based on the basic concept in^27^. The resistance of the periodically structured microstrip line, which represents the ohmic dissipation, can be calculated by using the perturbation method. Th dissipation power *P*_*d*_ in metal per unit cell can be written as

3 $${P}_{d}=\frac{1}{2}{R}_{s}{{\iint }_{s}|{H}_{t}|}^{2}ds$$

where the integral is made over all the metal surface in the unit cell, *R*_*s*_ is real part of the metal surface resistance, and *H*_*t*_ is the tangential component of the magnetic field on the metal surface. Then the resistance *R*_*d*_ is defined by

4 $${P}_{d}=\frac{1}{2}{R}_{d}{|I|}^{2}$$

where *I* is the total current on the microstrip line surface and it can also be obtained from *H*_*t*_ on the metal surface. The fields of SSPPs are obtained by using commercial (COMSOL Multiphysics) software through solving the eigenfrequency problem. In the numerical calculation, only a unit cell of the periodically structured microstrip line is considered. A periodic boundary condition (with the Bloch wavevector) is employed in the propagation direction, and the computation domain is terminated by perfectly matched layer in the other directions.

From the simulated electric field of SSPPs, we calculate the voltage between the signal line and the ground plane. The charges in the signal line is also calculated. The capacitance is evaluated according to the basic definition. It is known that *RLGC* equivalent circuit can effectively describe the transmission property of an infinitesimal section of uniform transmission line^28^. Since the unit cell of our periodically structured microstrip line is much smaller than the wavelength, it should be very effective to employ the circuit model to describe it. This will be verified subsequently by our numerical simulation and TDR measurement. The circuit models for our structured microstrip lines will be used to calculate the *S*-parameters for wave transmission.

**Figure 3:**
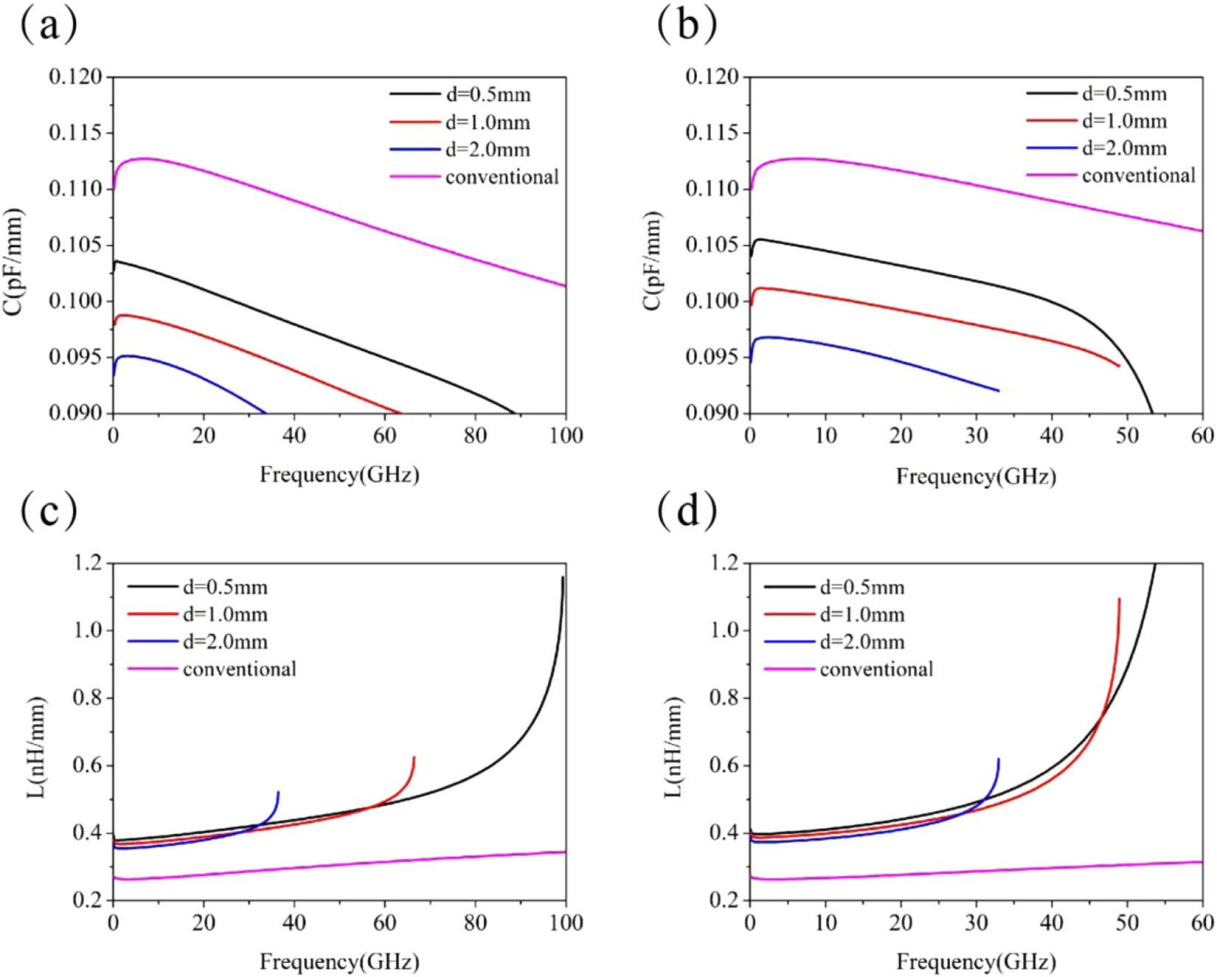
Capacitances and inductances per unit length for different periods. (**a,c**) correspond to the numerical results for the microstrip lines with BSPCs, (**b,d**) correspond to the numerical results for the microstrip lines with USPCs. For comparison, the corresponding results for the conventional microstrip line are included.

The calculated capacitances per unit length for our structured microstrip lines at frequencies below the asymptotic frequency *f*_*s*_ are plotted in Fig. 3a,b, and for comparison, the results for corresponding conventional microstrip line are also included. For each type of periodically structured microstrip lines, three lattice constants of *d* = 0.5, 1.0, and 2.0 mm are considered. Figure 3(a) shows the results for the microstrip lines with BSPCs, while Fig. 3(b) shows the results for the microstrip lines with USPCs. For the conventional microstrip line, the capacitance per unit length is *C* = 0.11004(pF/mm) at *f* = 0.1838 GHz and reaches a maximum of *C* = 0.11272(pF/mm) at *f* = 6.225 GHz, then it slowly decreases as *f* grows. At *f* = 100 GHz, the capacitance per unit length is reduced to *C* = 0.10129 (pF/mm). For both types of the structured microstrip lines, the dependence of the capacitance per unit length on the frequency is similar to that for the conventional microstrip line, but they reach their maximal values at lower frequencies. Due to the presence of high-density grooves in the metal strip, the capacitance for the structured microstrip lines is always smaller than that for the conventional microstrip line. For the BSPC microstrip line *d* = 0.5 mm, the capacitance per unit length has a maximal value of *C* = 0.10361(pF/mm) at *f* = 0.638 GHz, and it drops to *C* = 0.08587(pF/mm) at *f* = 99.25 GHz, which are smaller than the corresponding values for the conventional microstrip line. It can be found that the capacitance decreases when the lattice constant increases. The maximal value of the capacitance per unit length is *C* = 0.09878(pF/mm) at *f* = 1.991 GHz for *d* = 1.0 mm, and *C* = 0.09514 (pF/mm) at *f* = 2.58 GHz for *d* = 2.0 mm. The capacitance for the microstrip line with USPCs behaves like that for the microstrip line with BSPCs. In the case of *d* = 0.5 mm, the capacitance has a highest value of *C* = 0.10553 (pF/mm) at *f* = 1.236 GHz and it decreases to *C* = 0.0677(pF/mm) at *f*_*s*_ = 59.34 GHz. For the case of *d* = 1.0 mm and *d* = 2.0 mm, it has the highest capacitance values of *C* = 0.1012 (pF/mm) at *f* = 1.597 GHz and *C* = 0.09681 (pF/mm) at *f* = 2.493 GHz.

Figure 3(c,d) show the inductances per unit length for our structured microstrip lines, and the results for the corresponding conventional microstrip line are also included for comparison. The inductances for the microstrip line with BSPCs are displayed in Fig. 3(c) and three lattice constants of $d=0.5$, $1.0$, and $2.0$ mm are analyzed. For the conventional microstrip line, the inductance (per unit length) increases with frequency, from minimal value $L=0.26362$(nH/mm) at $f=2.938$ GHz to $L=0.34497$ (nH/mm) at $f=100$ GHz, which only has small change in the considered frequency range. For the BSPC microstrip line with $d=0.5$ mm, the inductance (per unit length) is $L=0.3791$ (nH/mm) at $f=0.637$ GHz, and it slowly grows to $L=0.4807$(nH/mm) at $f=58.39$ GHz. But when the frequency is close to ${f}_{s}$, it quickly increases and reaches $1.15$(nH/mm) at $f=99.3$ GHz. The inductance (per unit length) in the low-frequency band is nearly $1.43$ times larger than that for the conventional microstrip line, but in the vicinity of $f=100$ GHz, the former becomes $3.3$ times larger than the latter. In the case when $d=1.0$ mm, the inductance is $L=0.3678$ (nH/mm) at $f=1.6592$ GHz and it slowly increases to $L=0.624$ (nH/mm) at ${f}_{s}=66.46$ GHz,; in the case of $d=2.0$ mm, the inductance is $L=0.355$ (nH/mm) at $f=1.893$ GHz and it increases to $L=0.521$ (nH/mm) at ${f}_{s}=36.44$ GHz. Evidently, the microstrip line with BSPCs always has a larger inductance than the conventional microstrip line. This is the same situation for the other structured microstrip line with USPCs, and the similar results are displayed in Fig. 3(d). For the USPC microstrip line with $d=0.5$ mm, the inductance (per unit length) is $L=0.397$(nH/mm) at $f=1.236$ GHz, and it slowly grows to $L=1.0078$(nH/mm) at $f=51.75$ GHz, but it rapidly increases to $L=4.1516$(nH/mm) at ${f}_{s}=59.34$ GHz. Obviously, the microstrip line with USPCs has larger inductance than the one with BSPCs, especially at frequencies near *f*_*s*_.

**Figure 4:**
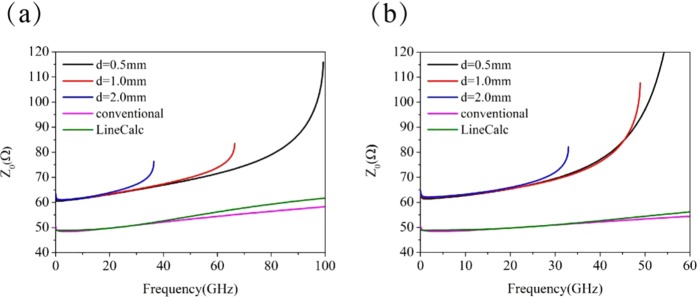
The characteristic impedances of the microstrip lines with (**a**) BSPCs and (**b**) USPCs.

With the knowledge of capacitance and inductance per unit length, we are now able to analyze the characteristic impedance and transmission properties of the two types of periodically structured microstrip lines. The characteristic impedances for both the structured microstrip lines are plotted in Fig. 4, and for comparison, the results for the conventional microstrip line are also included. Figure 4(a) displays the characteristic impedances for the microstrip line with BSPCs, and three cases of $$d=0.5$$, $$1.0$$, and $$2.0$$ mm are considered. For the conventional microstrip line, the characteristic impedance increases from $${Z}_{0}=48.54\Omega $$ at $$f=1.47$$ GHz to $${Z}_{0}=58.36\,\Omega $$ at $$f=100$$ GHz, which is almost the same as the value obtained from LineCalc in ADS. The characteristic impedance for the microstrip line with BSPCs is much higher than that for the conventional microstrip line, due to the existence of periodic corrugations. In the case of $$d=0.5$$ mm, it slowly increases from $${Z}_{0}=60.493$$$$\Omega $$ at $$f=0.638$$ GHz to $${Z}_{0}=70.22\,\Omega $$ at $$f=55$$ GHz, and then it quickly increases to $${Z}_{0}=115.92\,\Omega $$ at the SSPP asymptotic frequency of $$99.3$$ GHz. The variation range of the characteristic impedance is small within the low frequency band, which makes the structured microstrip line suitable for application in practical circuit. In the case when *d* = 1.0 mm, the characteristic impedance is $${Z}_{0}=61.02$$ $$\Omega $$ at $$f=1.99$$ GHz and increases to $${Z}_{0}=83.46$$ $$\Omega $$ at $${f}_{s}=66.46$$ GHz, and for the case of $$d=2.0$$ mm, $${Z}_{0}=61.09$$ $$\Omega $$ at $$f=1.893$$ GHz and increases to $${Z}_{0}=76.39$$$$\Omega $$ at $${f}_{s}=36.44$$ GHz. It is worth mentioning that the characteristic impedances for the different *d* values are almost the same at low frequencies. But at the asymptotic frequency, the characteristic impedance $${Z}_{0}$$ has a larger value for smaller *d*.

Figure 4(b) presents the characteristic impedance for the microstrip lines with USPCs for the cases of $d=0.5$, 1.0 and 2.0 mm. Compared with the conventional microstrip line, the characteristic impedance of this structured microstrip line increases much faster with frequency. In the case of $d=0.5$ mm, the characteristic impedance for the microstrip lines with USPCs slowly grows from ${Z}_{0}=61.34$ $\Omega$ at $f=1.23$ GHz to ${Z}_{0}=71.36$ $\Omega$ at $f=33.19$ GHz, and then quickly increases to ${Z}_{0}=247.59\,\Omega$ at ${f}_{s}=59.34$ GHz. Like the microstrip line with BSPCs, the microstrip lines with USPCs for the different *d* values almost have the same characteristic impedances at low frequencies, and they have remarkably different values at their asymptotic frequencies. For the case of $d=1.0$ mm, the characteristic impedance is ${Z}_{0}=107.62$ $\Omega$ at ${f}_{s}=48.92$ GHz, and it becomes ${Z}_{0}=82.16\,\Omega$ at ${f}_{s}=32.95$ GHz for $d=2.0$ mm.

**Figure 5:**
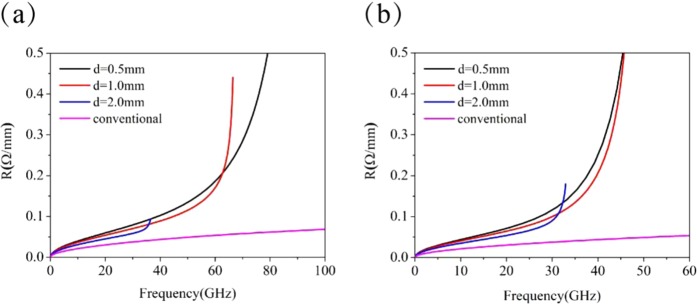
The resistance per unit length for the microstrip lines with (**a**) BSPCs and (**b**) USPCs.

Figure 5 shows the resistance per unit length for both the microstrip lines with BSPCs (a) and USPCs (b). Compared with the conventional microstrip line, the structured microstrip lines can confine the EM field more strongly, and thus it has a higher energy dissipation in the metal. Consequently, the structured microstrip lines have a larger resistance than the conventional one. Furthermore, the resistance increases quickly with frequency, and it is larger for smaller *d*. As the microstrip line with USPCs is more effective in confining field than the one with BSPCs, it has larger resistance than the latter. However, in low frequency band, there is no difference in the resistance between the microstrip lines with USPCs and BSPCs. The increase of resistance with frequency will cause the high frequency components of the digital signal to decay rapidly. The rising time of the digital signal will become longer when employing the structured microstrip lines. Since the microstrip line with BSPCs has significantly smaller resistance than the one with USPCs in the high frequency range, it can provide larger frequency bandwidth and has less influence on the rising time of the signal.

It is pointed out in^22^ that the dielectric losses are greater than the conductor losses at frequencies above 1 GHz. For high-speed serial links with clock frequencies at or above 2.5 Gbps, the dielectric losses dominate. The conductance per unit length *G* of the microstrip line is always neglected when extracting the equivalent circuit parameters, which causes the S-parameters from the equivalent circuit model to deviate from the results from the full wave simulation. In addition, the conductance is proportional to the frequency for a fixed loss tan*δ*, thus there is severe deviation at both low and high frequencies when it is kept constant in the equivalent circuit. The conductance *G* can be determined from the formula^25^ as below

5 $$G=\frac{\omega \varepsilon ^{\prime\prime} }{{|{V}_{0}|}^{2}}{\iiint }_{v}\overrightarrow{E}\cdot {\overrightarrow{E}}^{\ast }dv$$

where *V*_0_ is the electric voltage obtained from the field integration between signal strip and ground, and $\varepsilon ^{\prime\prime}$ is the imaginary part of the dielectric permittivity. The volume integral for the square of the electric field amplitude is performed over the dielectric substrate. Our numerical analysis shows that *G* is almost linearly proportional to the frequency for both the microstrip lines with BSPCs and USPCs. In the numerical calculations, we take tan*δ* to be 0.0027. For the BSPC microstrip line with $d=1.0$ mm, *G* is found to be 0.00144 (S/m) at 1 GHz and 0.0145 (S/m) at 10 GHz. For the USPC microstrip line with *d* = 1.0 mm, *G* is 0.00148 at 1 GHz and 0.0149 (S/m) at 10 GHz.

**Figure 6:**
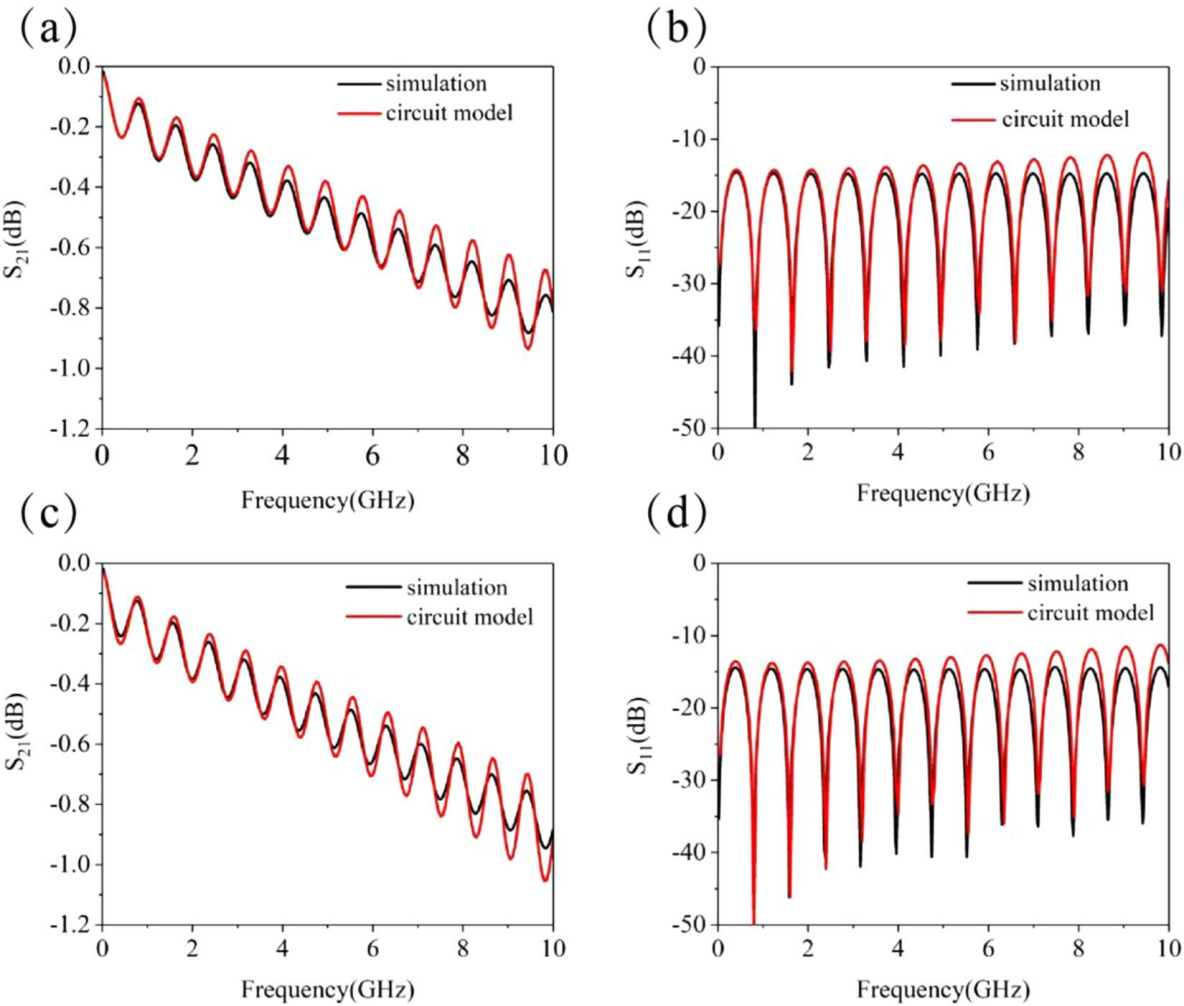
S-parameters of two structured microstrip lines with *d* = 1.0 mm characterized by the full wave simulation and equivalent circuit model. (**a**)*S*_21_ for the BSPC structure, (**b**) *S*_11_ for the BSPC structure, (**c**) *S*_21_ for the USPC structure, and (**d**) *S*_11_ for the USPC structure.

In order to verify the validity of the equivalent circuit parameters extracted from the numerical method, the equivalent circuit model is used for each unit cell of the subwavelength periodic microstrip line to calculate the S-parameters and compare it with the results from the full wave simulation. Figure 6 are the S-parameters for the microstrip lines with BSPCs and USPCs from both the equivalent circuit models and full wave simulations, where *d* = 1.0 mm. The total length of both the structured microstrip lines is taken to be 10 cm in the numerical calculations. It can be seen that the results obtained from the two different methods are in good agreement for the both structured microstrip lines.

### Experimental measurement

The measurement of characteristic impedance of conventional microstrip line often requires the use of a time domain reflectometer (TDR), and it is also clear that TDR can only provide the characteristic impedance for the transmission line at much low frequencies^29^. Although TDR can only provide a single value of characteristic impedance, and the conventional microstrip line is actually a waveguide with dispersion, but in most cases, it offers an important reference for PCB layout design. This is clearly indicated in the literature^30^ that the characteristic impedance of the microstrip line varies with frequency. To verify the validity of the numerically calculated characteristic impedance, we measured the impedance of the two structured microstrip lines with the TDR method, in which step pulse with a rising time of 30 ps and amplitude of 200 mV is used as excitation. The length of the structured microstrip lines under test is 10 cm.

**Figure 7:**
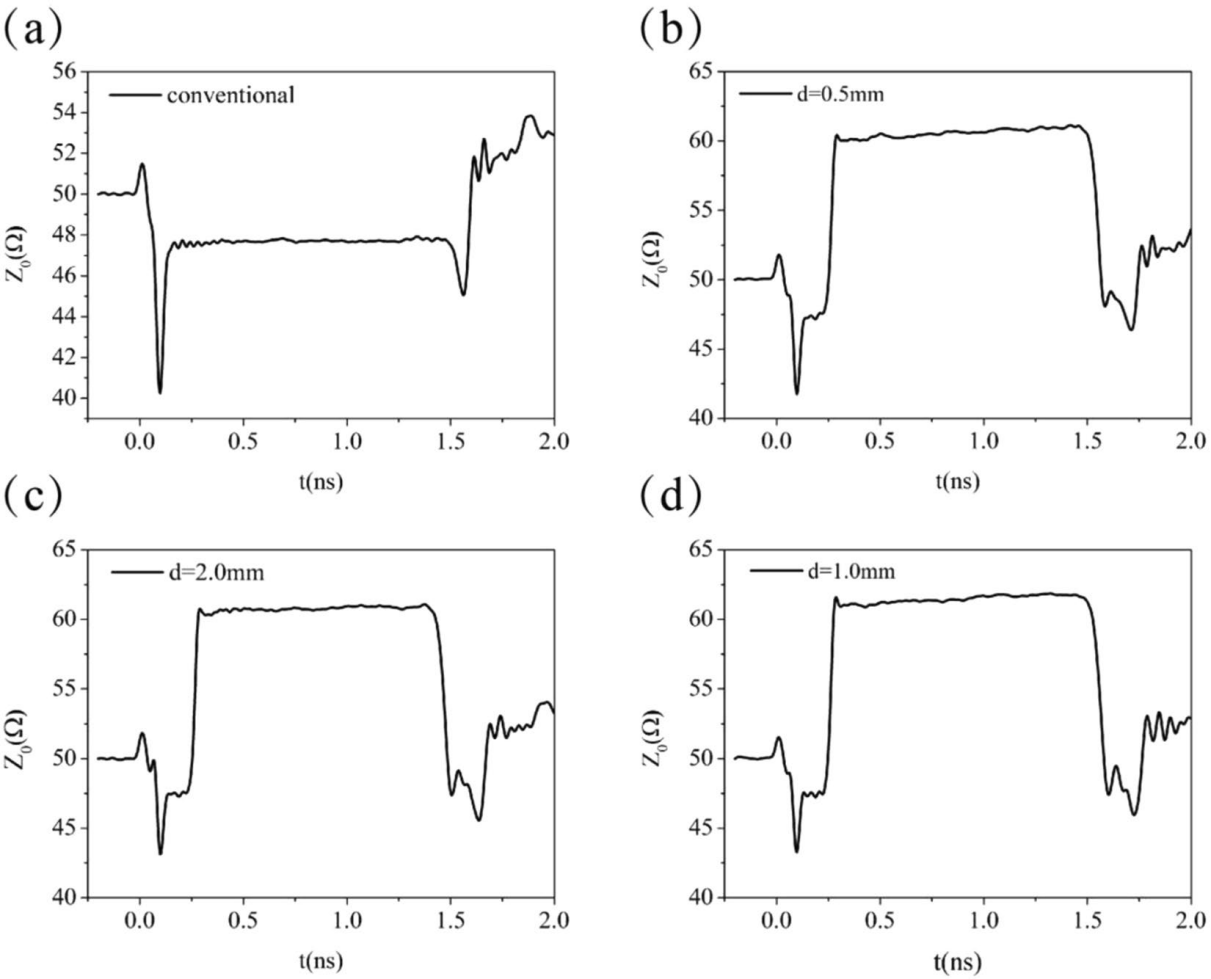
Measured impedances with TDR method for (**a**) the conventional microstrip line, (**b**) the BSPC microstrip line with *d* = 0.5 mm, (c) the BSPC microstrip line with *d* = 2.0 mm, and (d) the USPC microstrip line with *d* = 1.0 mm.

The impedance from TDR measurement is displayed in Fig. 7 as a function of time. Figure 7(a) shows the impedance of the conventional microstrip line, and in the time interval from 0.2423 to 1.489 ns, the measured value is between $47.49-47.831$ Ω, which is much close to the numerical value of ${Z}_{0}=48.54\,\Omega$ at much low frequency, the relative error is within 1.75%. The TDR measured impedance for the BSPC microstrip line with $d=0.5$ mm is ${Z}_{0}=60.6$ $\Omega$ at $t=1.0$ ns, as shown in Fig. 7(b), which is close to the minimal numerical value of ${Z}_{0}=60.493\,\Omega$, the relative error is only within 0.17%. The measured impedance is located between $60.49\,\Omega$ and $61.139\,\Omega$ over the time interval from 0.29 to 1.497 ns, where the curve of impedance versus time is almost a horizontal line, as in the situation for the conventional microstrip line. Therefore, a microstrip line with subwavelength periodic corrugations can be effectively described by a uniform microstrip line with a different characteristic impedance. This is an extremely important result. In addition, the variation of characteristic impedance obtained from the theoretical calculations is less than 7% when the frequency is below 20 GHz. All these eliminate the doubt if the concept of SSPPs can be directly used in the actual circuit. Figure 7(c) shows the TDR measured impedance for the BSPC microstrip line with $d=2.0$ mm. We find that ${Z}_{0}=60.96\,\Omega$ at $t=1.0$ ns, while the minimal numerical value is ${Z}_{0}=61.09\,\Omega$. This means that the measured value has an accuracy within 0.21%. Besides, the experiencing time of signal in the structured microstrip line is different for different lattice constants, and it is longer for a shorter lattice constant. Figure 7(d) shows the impedance of the USPC microstrip line with $d=1.0$ mm. The impedance is ${Z}_{0}=61.7$ $\Omega$ at $t=1.0$ ns, which is close to the calculated value ${Z}_{0}=61.86\,\Omega$, and the relative error is only 0.25%.

**Figure 8:**
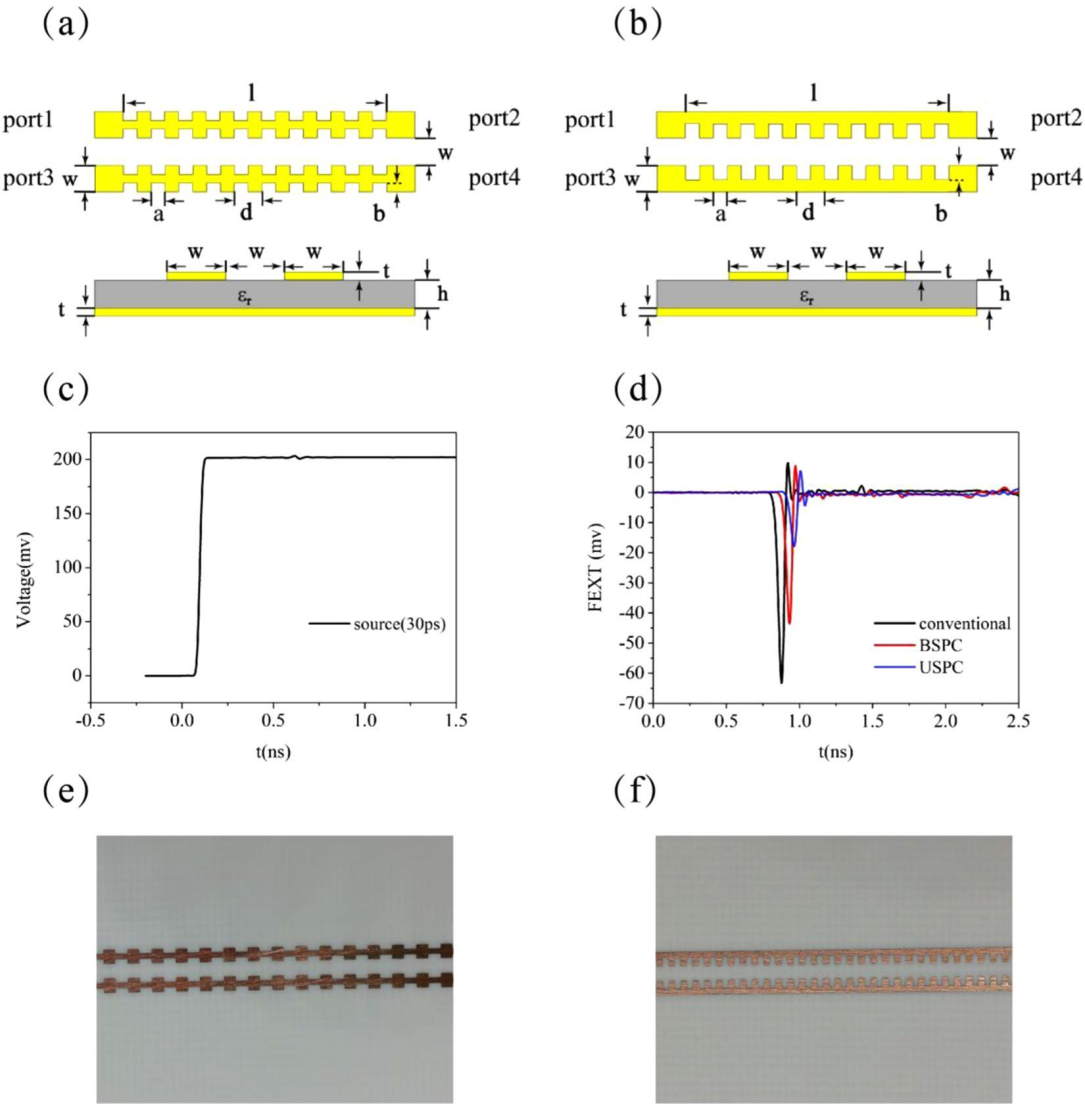
Mutual coupling effect between two parallel microstrip lines with (**a**) BSPCs or (**b**) USPCs. (**c**) The input signal at port 1. (**d**) The measured FEXT. (**e**) Optical image of the coupled BSPC microstrip lines. (**f**) Optical image of the coupled USPC microstrip lines.

To demonstrate experimentally that the periodically structured microstrip lines have the ability to suppress mutual coupling between them, we consider the coupling circuits illustrated in Fig. 8(a,b), where a pair of two BSPC or USPC microstrip lines are separated by a distance of $w=1.2$ mm. For both types of the structured microstrip lines, the lattice constant is $d=1.0$ mm, the substrate thickness is $h=0.508$ mm and its dielectric constant ${\varepsilon }_{r}=3.37$. The groove depth is $b=0.3w$ for BSPCs and $b=0.6w$ for USPCs. The coupling length is $l=10$ cm. In the experiment, we inject a step pulse signal into port 1 of the coupled structured microstrip lines with a rising time of 30 ps and amplitude of 200 mV, as shown in Fig. 8(c).

The far end cross-talk (FEXT) at port 4 is shown in Fig. 8(d). It can be found that the peak value of coupling signal is −63.28 mV for the conventional microstrip line, and it can be reduced to −43.47 mV for the BSPC system and −17.84 mV for the USPC system. For the coupled UPSC microstrip lines, the FEXT is only 28.2% of that for the conventional microstrip lines, while it is 68.7% for the coupled BPSCs microstrip lines. For the circuit system that urgently needs to suppress the FEXT, the UPSC structure is a good candidate for the transmission line. Obviously, the UPSC structure with subwavelength periodic corrugations along its edge can effectively squeeze the electromagnetic field into the grooves, thus it can effectively suppress the coupling with an adjacent microstrip line. It is apparent that SSPPs can be applied in suppressing mutual coupling in high density circuit.

## Conclusion

In this paper, we have calculated the equivalent circuit parameters (such as inductance, capacitance, resistance and conductance) for microstrip line with subwavelength periodic corrugations, and thus obtained its characteristic impedance. TDR measurement reveals that the structured microstrip line has a constant value of characteristic impedance, and it is highly consistent with the lowest value of the characteristic impedance of the numerical results. The S-parameters obtained from the equivalent circuit model with the extracted circuit parameters has been found to agree well with those from the full wave simulation by the commercial software. This confirms that the metamaterial can be directly applied to the actual circuit, and we provide the characteristic impedance of the the structured microstrip line for circuit designers. The experimental results of the actual circuit show that the subwavelength periodic microstrip line has an excellent anti-interference characteristic.

## Methods

### Experimental measurement

The measurement of characteristic impedance of the conventional microstrip line often requires the use of a time domain reflectometer (TDR). Although TDR can only provide a single value of characteristic impedance, and the conventional microstrip line is a waveguide with dispersion, in most cases, it offers an important reference value for PCB layout design. Therefore, the characteristic impedance of subwavelength corrugated microstrip lines is measured using conventional TDR.

### Simulation set-up

The modal fields of subwavelength corrugated microstrip line are calculated with the first-principles electromagnetic simulation software COMSOL Multiphysics. According to their definitions, the equivalent circuit parameters of the proposed microstrip line are further calculated from the simulated fields.
